# Machine learning approaches to predict gestational age in normal and complicated pregnancies via urinary metabolomics analysis

**DOI:** 10.1038/s41598-021-97342-z

**Published:** 2021-09-07

**Authors:** Takafumi Yamauchi, Daisuke Ochi, Naomi Matsukawa, Daisuke Saigusa, Mami Ishikuro, Taku Obara, Yoshiki Tsunemoto, Satsuki Kumatani, Riu Yamashita, Osamu Tanabe, Naoko Minegishi, Seizo Koshiba, Hirohito Metoki, Shinichi Kuriyama, Nobuo Yaegashi, Masayuki Yamamoto, Masao Nagasaki, Satoshi Hiyama, Junichi Sugawara

**Affiliations:** 1 X-Tech Development Department, NTT DOCOMO, INC, 3-6 Hikarino-oka, Yokosuka, Kanagawa 239-8536 Japan; 2 Tohoku Medical Megabank Organization, Tohoku University, 2-1 Seiryo-machi, Aoba-ku, Sendai, 980-8573 Japan; 3 Tohoku University Graduate School of Medicine, Tohoku University, 2-1 Seiryo-machi, Aoba-ku, Sendai, 980-8575 Japan; 4 Exploratory Oncology Research and Clinical Trial Center, National Cancer Center, 6-5-1 Kashiwanoha, Kashiwa, Chiba 277-8577 Japan; 5 Radiation Effects Research Foundation, 5-2 Hijiyama Park, Minami-ku, Hiroshima, 732-0815 Japan; 6 Advanced Research Center for Innovations in Next-Generation Medicine, Tohoku University, 2-1 Seiryo-machi, Aoba-ku, Sendai, 980-8573 Japan; 7 Faculty of Medicine, Tohoku Medical Pharmaceutical University, 4-4-1 Komatsushima, Aoba-ku, Sendai, 981-0905 Japan; 8 International Research Institute of Disaster Science, Tohoku University, Aramaki Aza-Aoba 468-1, Aoba-ku, Sendai, 980-8572 Japan; 9 Center for Genomic Medicine, Graduate School of Medicine, Kyoto University, 53 Shogoinkawahara-cho, Sakyo-ku, Kyoto City, Kyoto 606-8507 Japan; 10 Center for the Promotion of Interdisciplinary Education and Research, Kyoto University, Yoshida-Honmachi, Sakyo-ku, Kyoto, 606-8507 Japan

**Keywords:** Predictive markers, Metabolomics

## Abstract

The elucidation of dynamic metabolomic changes during gestation is particularly important for the development of methods to evaluate pregnancy status or achieve earlier detection of pregnancy-related complications. Some studies have constructed models to evaluate pregnancy status and predict gestational age using omics data from blood biospecimens; however, less invasive methods are desired. Here we propose a model to predict gestational age, using urinary metabolite information. In our prospective cohort study, we collected 2741 urine samples from 187 healthy pregnant women, 23 patients with hypertensive disorders of pregnancy, and 14 patients with spontaneous preterm birth. Using gas chromatography-tandem mass spectrometry, we identified 184 urinary metabolites that showed dynamic systematic changes in healthy pregnant women according to gestational age. A model to predict gestational age during normal pregnancy progression was constructed; the correlation coefficient between actual and predicted weeks of gestation was 0.86. The predicted gestational ages of cases with hypertensive disorders of pregnancy exhibited significant progression, compared with actual gestational ages. This is the first study to predict gestational age in normal and complicated pregnancies by using urinary metabolite information. Minimally invasive urinary metabolomics might facilitate changes in the prediction of gestational age in various clinical settings.

## Introduction

Pregnancy induces dynamic and temporal changes in maternal physiological profiles^1^. Recent reports suggested that various omics profiles (e.g., those of transcriptomics, epigenomics, metabolomics, and microbiomics) show substantial temporal changes during pregnancy^2–5^. Furthermore, changes in omics profiles are associated with pregnancy complications^6,7^.

To evaluate pregnancy status and predict pregnancy complications, several groups have attempted to characterize normal pregnancy via omics analyses of maternal biospecimens^8–10^. Specifically, Liang and colleagues have constructed models to predict gestational age based on plasma metabolites at the time of specimen collection^11^. The models could predict gestational age with high accuracy (i.e., correlation coefficient of 0.92 between predicted and actual gestational ages); those predictive models precisely reflected physiological adaptations in pregnant women. However, the models thus far have used omics information collected from maternal blood. Furthermore, there have not been investigations regarding whether predictive models show distinct results in cases with pregnancy complications, compared with women who have normal pregnancy. Therefore, less invasive methods are desired for the prediction of gestational age in women with normal and complicated pregnancies. Several studies have shown that urinary metabolome information is associated with blood metabolome information^12,13^. Moreover, urine can be collected in a minimally invasive manner at each routine antenatal visit in most clinical settings^14^.

The present study examined the dynamic urinary metabolomic profiles during pregnancy, then proposed a model to predict gestational age by means of urinary metabolomics analysis. Furthermore, by applying the predictive model to urinary metabolite data from women who subsequently developed pregnancy complications, this study revealed changes in predicted gestational ages, compared with actual ages, in women with complicated pregnancies.

## Results

### Maternal clinical backgrounds

Clinical backgrounds of study participants are shown in Table 1. Among the healthy pregnant women, the mean maternal age at the 40th gestational week and the mean pre-pregnancy body mass index were 32.7 ± 4.8 years, and 22.2 ± 4.7 kg/m^2^, respectively. Of the healthy cases, 51% were parous. The mean (± standard deviation) gestational age at delivery was 38.6 ± 1.2 weeks.

**Table 1:** Clinical characteristics of pregnant women in this study.

Variable	Healthy (n = 187)	HDP (n = 23)	*p*-value*	SPTB (n = 14)	*p*-value*
Age at 40th gestational week, years	32.7 (± 4.8)	35.5 (± 6.1)	0.045	34.1 (± 4.9)	0.33
Pre-pregnancy body mass index, kg/m^2^	22.2 (± 4.7)	22.4 (± 3.6)	0.78	21.4 (± 4.0)	0.50
Parity	51%	39%	0.38	57%	0.78
Gestational age at delivery, weeks	38.6 (± 1.2)	37.5 (± 2.0)	0.017	35.1 (± 1.4)	< 0.0001

*Categorical or continuous variables were compared between the healthy group and either HDP or SPTB groups using Fisher's exact test or Welch’s t-test, respectively. Regarding gestational age at delivery, significant differences (*p* < 0.05) were observed using the Mann–Whitney U test. Data are presented as the mean ± S.D.

Compared with healthy pregnant women, the maternal age was higher among subjects with hypertensive disorders of pregnancy (HDP), while the gestational ages at delivery were lower in subjects with HDP or spontaneous preterm birth (SPTB). No other variables significantly differed between healthy pregnant women and subjects with complications.

### Urinary metabolite profiles during normal pregnancy

**Figure 1:**
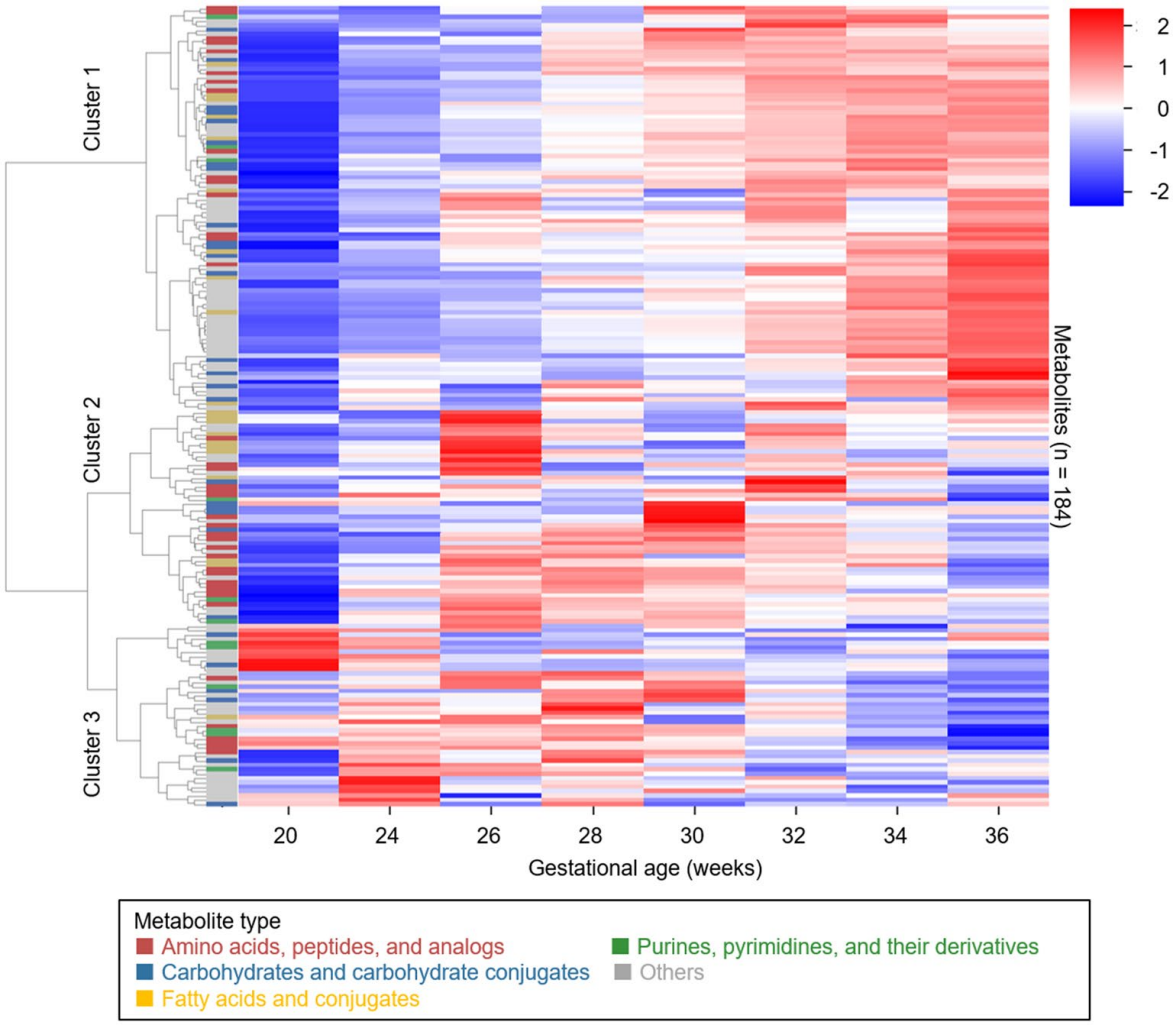
Changes in urinary metabolite levels during normal pregnancy. Levels of metabolites in urine samples from 187 healthy pregnant women were averaged for each gestational week, standardized by the z-score method, and presented as a heatmap. On the left, metabolite types are indicated using red, blue, yellow, green, and gray stripes; these indicate “amino acids, peptides, and analogs,” “carbohydrates and carbohydrate conjugates,” “fatty acids and conjugates,” “purines, pyrimidines, and their derivatives,” and “others,” respectively.

**Figure 2:**
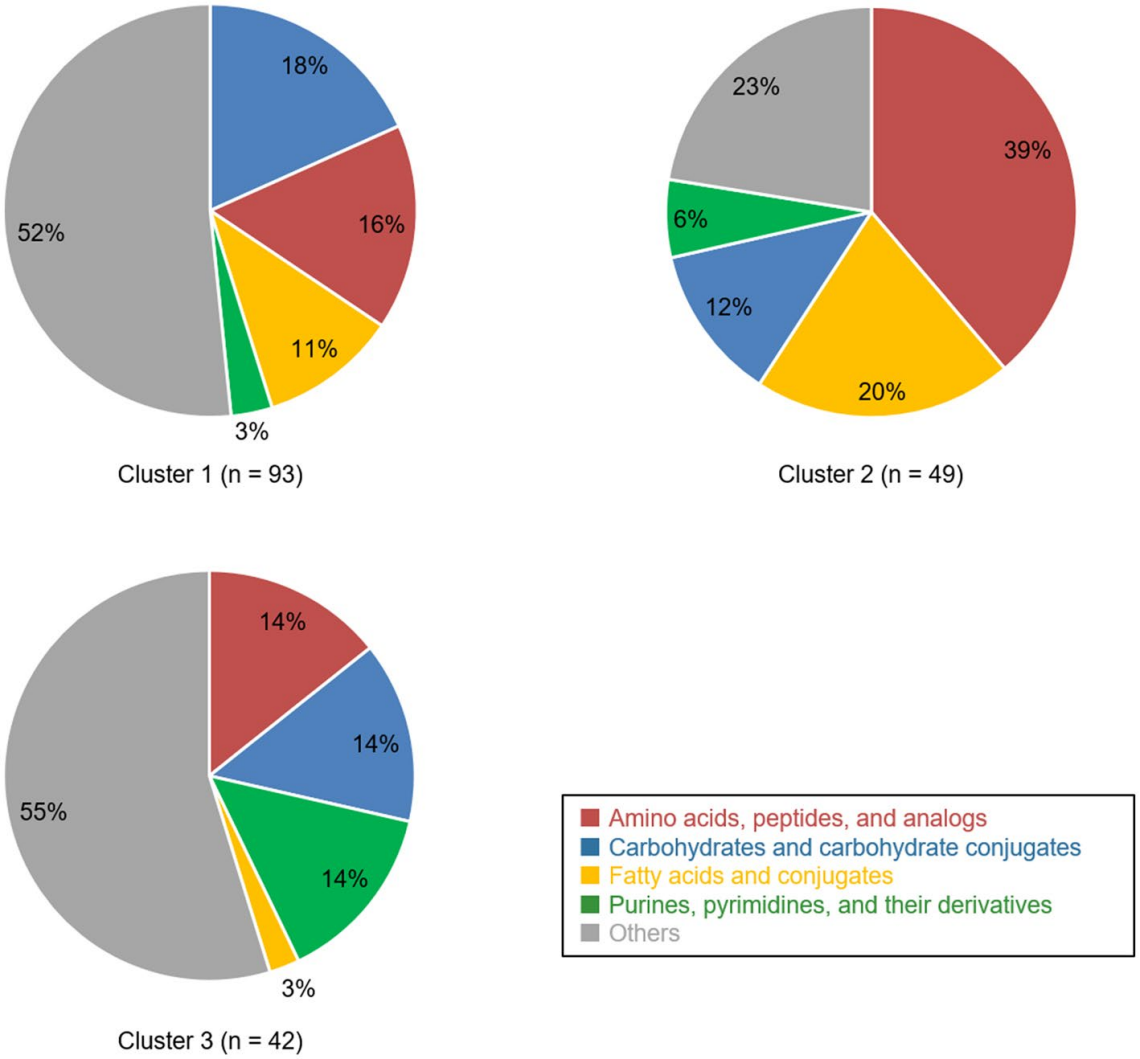
Proportions of metabolite types in each cluster. Red, blue, yellow, green, and gray sectors indicate “amino acids, peptides, and analogs,” “carbohydrates and carbohydrate conjugates,” “fatty acids and conjugates,” “purines, pyrimidines, and their derivatives,” and “others,” respectively.

We investigated urinary metabolomic profiles during normal pregnancy. Figure 1 shows hierarchical clustering of changes in the levels of urinary metabolites in healthy pregnant women (n = 187) during gestation. Relative levels of multiple metabolites changed dynamically according to gestational age. Approximately half of the metabolites were in cluster 1 (upper part of Fig. 1); the normalized levels of metabolites in this cluster increased with gestational age (correlation coefficient = 0.86). Cluster 1 included comparatively greater proportions of amino acids, peptides, and carbohydrates (Fig. 2, and Supplementary Table 1 online); for example, this cluster included sulfur-containing amino acids (e.g., cysteine and methionine) and monosaccharides (e.g., glucose and lactose). In contrast, the normalized levels of metabolites in cluster 3 were negatively correlated with gestational age (correlation coefficient = − 0.38). This cluster included greater proportions of purines and pyrimidines (e.g., cytosine, thymine, and uracil), compared with other clusters. Hydrophilic basic amino acids (e.g., histidine and lysine) were also present in this cluster. In contrast to metabolites in clusters 1 and 3, metabolites in cluster 2 did not show a clear linear relationship with gestational age (correlation coefficient = 0.24, see Supplementary Fig. 1 online). Comparatively greater proportions of amino acids and fatty acids (e.g., aromatic amino acids and stearic acid) were characteristic of cluster 2.

### Predictive model to estimate gestational age

**Figure 3:**
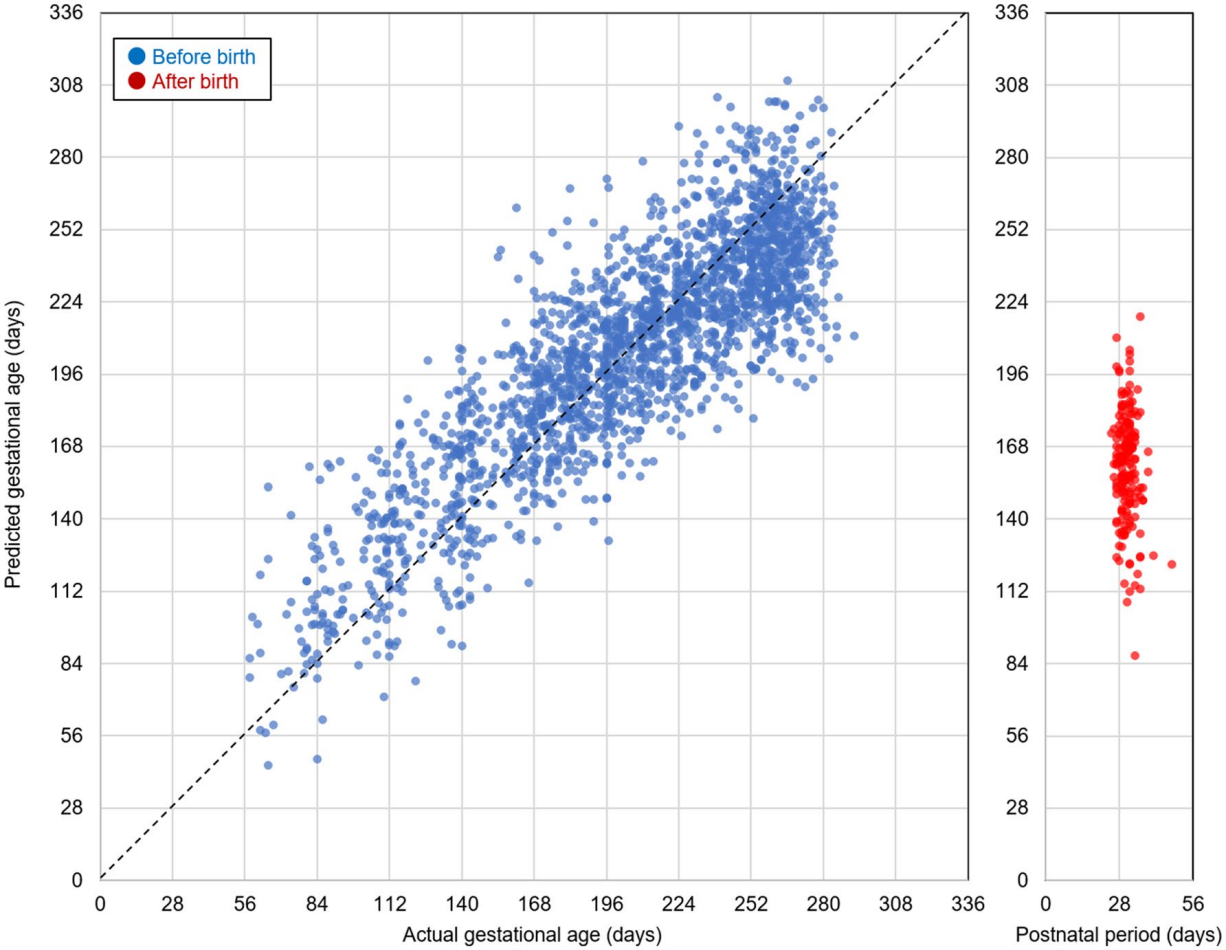
Prediction of gestational age in healthy pregnant women. Blue dots indicate actual days (horizontal axis) and predicted days (vertical axis) of gestation when leave-one-out cross validation was performed for each healthy pregnant woman using urine samples collected before delivery. Red dots indicate gestational ages estimated by the predictive model, which was trained with urine samples before delivery, using metabolite levels in samples collected after delivery.

Using urinary metabolites from healthy pregnant women, we constructed a predictive model to estimate gestational age at the time of urine collection. Blue dots in Fig. 3 show actual and predicted days of gestation when leave-one-out cross validation was performed for each healthy pregnant woman. The root mean squared error between the actual and predicted days of gestation was 26.7, while the Pearson correlation coefficient was 0.86.

Sixty-eight of the 184 metabolites consistently contributed to the predictive model; no metabolite coefficient reached zero during leave-one-out cross validation (see Supplementary Table 1 online). The top 10 metabolites with the greatest contributions to the predictive model are shown in Table 2. These contributions were evaluated by the absolute values of coefficients. Eight of these 10 metabolites were classified in either cluster 1 or 3 in Fig. 1. These results indicated that our predictive model successfully selected metabolites that were highly correlated with gestational age.

**Table 2:** Top 10 metabolites that contributed most to the predictive model.

Metabolite	Cluster	Coefficient
Threonine	1	0.27
7-Methylguanine	1	0.19
2-Hydroxyglutaric acid	1	0.17
Xanthine	3	− 0.17
Batyl alcohol	1	0.16
3-Hydroxy-3-methylglutaric acid	1	0.16
Acetylglycine	2	− 0.14
5-Oxoproline	3	− 0.13
Spermine	1	0.12
Arginine	2	− 0.11

The results of enrichment analysis for the 68 metabolites are shown in Table 3. In cluster 1, metabolites related to cysteine metabolism were enriched. The metabolism of homocysteine, which is located upstream of cysteine metabolism, is considered crucial for the maintenance of a healthy pregnancy^15^. Metabolites related to vitamin and purine metabolic processes were significantly enriched in cluster 3.

**Table 3:** Top five biofunctions for each cluster annotated by enrichment analysis of 68 metabolites.

Biofunction	*p*-value	False discovery rate
**Cluster 1**		
Components of cysteine metabolism	4.0e–10	1.8e–08
Components of arginine and proline metabolism	2.1e–08	4.9e–07
Components of nitrogen metabolism	2.9e–07	4.4e–06
Components of glutamate metabolism	2.1e–06	2.4e–05
Components of glycine, serine, and threonine metabolism	5.9e–06	5.5e–05
**Cluster 2**		
Components of glycine, serine, and threonine metabolism	1.9e–07	4.7e–06
Essential amino acids	2.9e–06	3.5e–05
Components of aminoacyl-tRNA biosynthesis	8.9e–06	5.3e–05
Components of cysteine metabolism	6.8e–06	5.3e–05
Semi-essential amino acids	5.4e–04	2.6e–03
**Cluster 3**		
Vitamins	1.0e–06	1.8e–05
Essential amino acids	9.1e–06	6.2e–05
Essential vitamins	1.0e–05	6.2e–05
Components of purine metabolism	7.3e–05	3.3e–04
Vitamins (Vitamin C)	6.6e–04	2.2e–03

Red dots in Fig. 3 indicate gestational age predicted by the model, using metabolite levels in urine samples collected after childbirth. Similar to the findings by Ghaemi et al., the predicted gestational age was significantly younger when our model was used with urine samples collected after childbirth than when our model was used with urine samples collected near delivery^10^.

### Prediction of gestational age in complicated pregnancies

**Figure 4:**
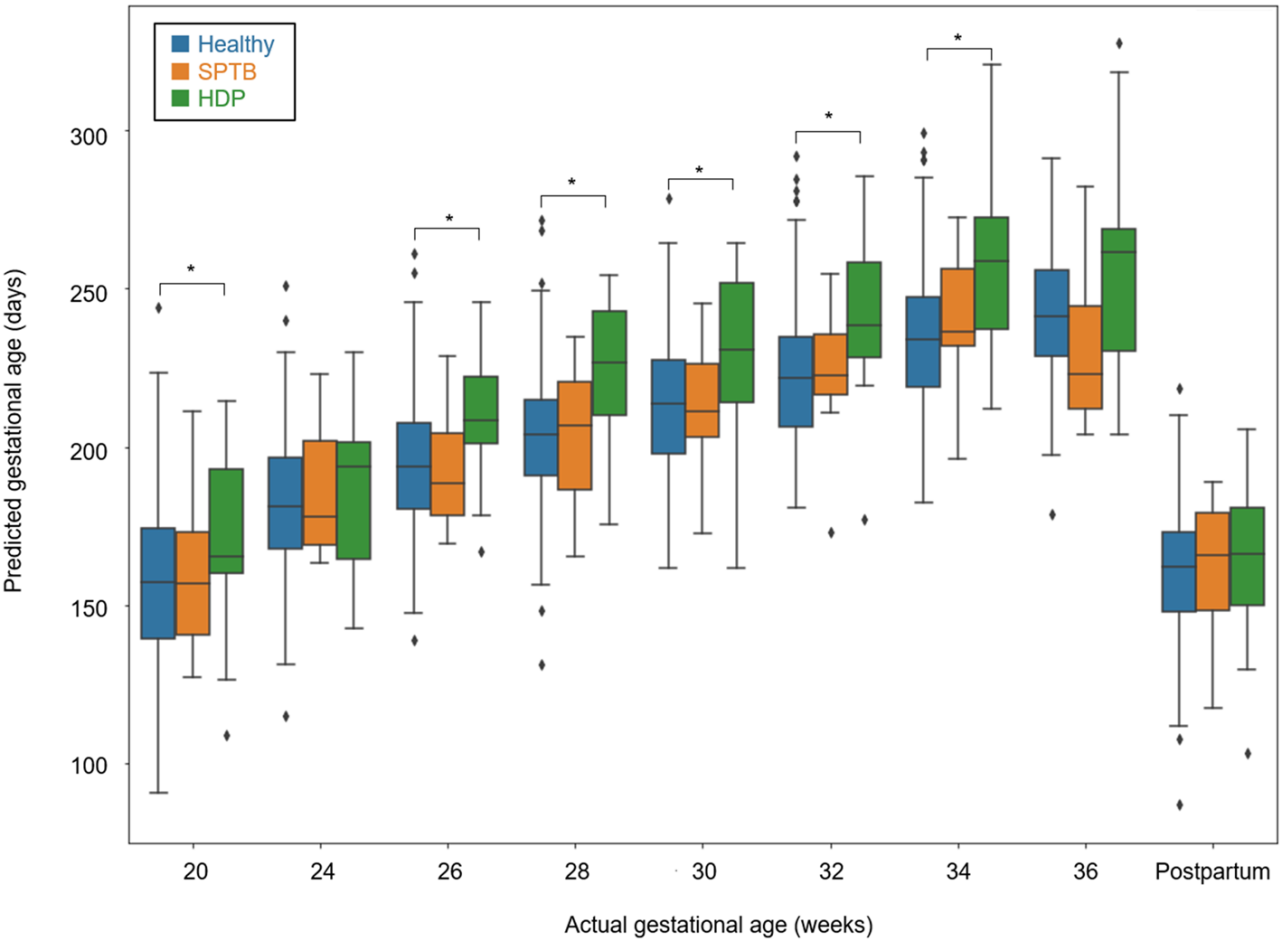
Comparison of predicted gestational ages among healthy, SPTB, and HDP cases. Asterisks indicate statistically significant differences by Welch’s t-test (*p* < 0.05) between healthy cases and those with pregnancy complications.

By entering urinary metabolite data from complicated pregnancies into the predictive model, we investigated potential differences in predicted gestational age between healthy and complicated pregnancies. After 20 weeks of gestation, the predicted gestational age was significantly older in HDP cases than in healthy pregnancies (Fig. 4). Comparison between healthy pregnancies and SPTB cases showed no significant differences in predicted gestational age or in metabolite levels (Fig. 4).

To analyze the effects of maternal age on the predictive model, we compared predicted ages between younger (< 35 years) and older (≥ 35 years) groups of women with normal pregnancies (see Supplementary Fig. 2 online). We found no significant differences between the two groups at any gestational age.

Subsequently, isocitric acid, 3-hydroxy-3-methylglutaric acid, and urocanic acid were identified as urinary metabolites that had significant associations with HDP onset (false discovery rate < 0.05) (see Supplementary Table 2 online). All three metabolites were classified in cluster 1 (Fig. 1); their urinary concentrations were higher in HDP cases than in healthy cases (see Supplementary Table 2 online).

**Figure 5:**
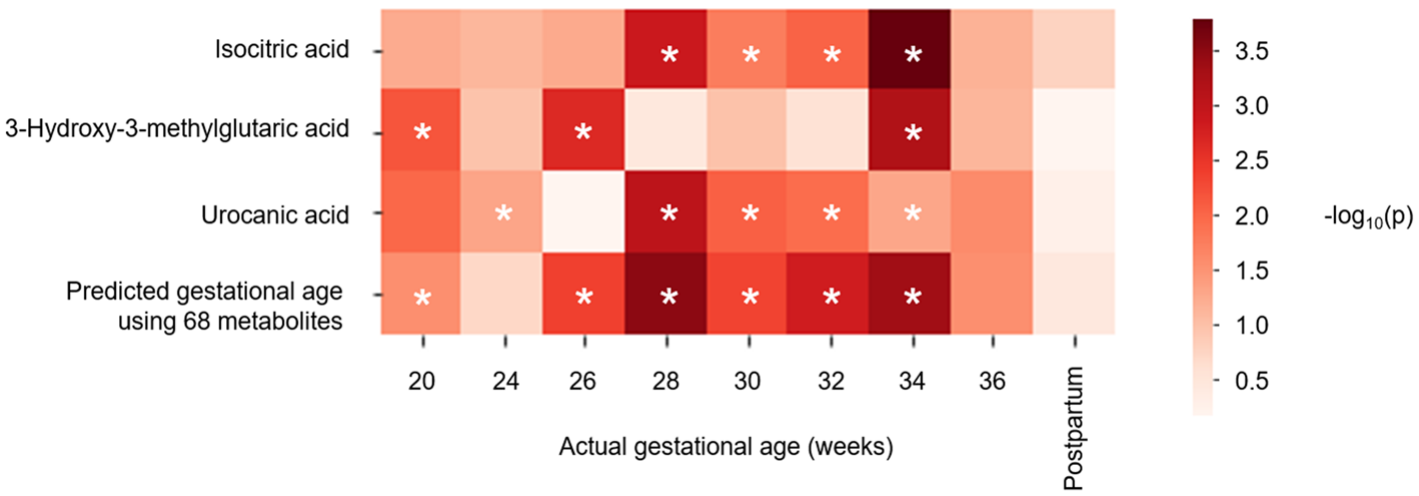
Statistical analyses of associations of urinary metabolites or predicted gestational age with HDP onset. Urinary levels of isocitric acid, 3-hydroxy-3-methylglutaric acid, and urocanic acid, as well as the predicted gestational age, were significantly associated with HDP onset at several weeks of gestation. *p*-values calculated by the Wald test, adjusted for maternal age and urine type, are shown as a heatmap for each gestational week. Asterisks in panel A indicate statistical significance (*p* < 0.05).

As shown in Fig. 5, the levels of these three metabolites and the predicted gestational age were significantly associated with HDP onset at several weeks of gestation. Compared with each of the three metabolites, the predicted gestational age was more strongly associated with HDP onset.

## Discussion

This study demonstrated dynamic changes in urinary metabolomic profiles during normal pregnancy. By means of machine learning algorithms, we constructed a predictive model that used urinary metabolite data to estimate gestational age during the progression of normal pregnancy. Moreover, we found that the predicted gestational age was significantly older than actual gestational age in cases with HDP.

This is a challenging attempt to predict gestational age in normal and complicated pregnancies by means of urinary metabolomics. Although a few studies have analyzed urinary metabolites in pregnant women, fewer than 50 metabolites were analyzed in each of those studies^16–18^.

We initially analyzed urine samples that were collected longitudinally from 187 healthy pregnant women, with the aim of elucidating urinary metabolite profiles during pregnancy. Hierarchical clustering showed that urinary metabolites comprised three distinct clusters (Fig. 1). The levels of metabolites contained in cluster 1, the largest cluster, increased with gestational age. These increased urinary metabolites during pregnancy might reflect the increased those in blood due to the reduction of maternal energy consumption associated with fetal and placental growth^19,20^. In a previous study, Diaz et al. used untargeted nuclear magnetic resonance-based metabolomics to examine temporal changes in the levels of 21 urinary metabolites during pregnancy; they identified 11 metabolites for which the levels significantly increased from the second trimester to the third trimester^21^. Three of those 11 metabolites (i.e., alanine, lactic acid, and threonine) were also identified in our study; all were included in cluster 1. Monosaccharides (e.g., glucose and lactose) were also included in cluster 1. The increased glucose level is consistent with the increased maternal insulin resistance that occurs during pregnancy^22^. A cohort study of 823 healthy pregnant women also showed similar results with respect to urinary lactose^23^. The increased lactose level was also present in blood metabolomics analyses; this was presumed to reflect maternal physiological changes to enable breastfeeding after delivery^24^.

Sixty-eight of the 184 metabolites with dynamic systematic changes consistently contributed to the predictive model; the coefficient of each metabolite never reached zero during leave-one-out cross validation. Therefore, these 68 metabolites are presumed to strongly reflect the progression of normal pregnancy. Enrichment analysis showed that 36 of the 68 metabolites (all in cluster 1) were related to cysteine metabolism. This suggests that homocysteine metabolism, which is located upstream of cysteine metabolism, is upregulated to maintain a healthy pregnancy^15^. Moreover, methionine, which is produced from homocysteine in the methionine cycle, was also classified in cluster 1, further supporting the potential upregulation of homocysteine metabolism.

The levels of metabolites classified in cluster 3 exhibited downward trends during pregnancy. This cluster included pyrimidines (e.g., cytosine, thymine, and uracil) and hydrophilic basic amino acids (e.g., histidine and lysine). Only a few previous studies have described the roles of, or changes in, these metabolites during pregnancy^18,25^. Furthermore, enrichment analysis of metabolites selected by the predictive model revealed significant enrichment of metabolites related to vitamins in cluster 3. Water-soluble vitamins (e.g., B_6_, B_12_, and C) play critical roles during pregnancy. For example, vitamin B_12_ is an important cofactor for DNA synthesis; it also participates in metabolic processes involving amino acids and fatty acids^26^. During pregnancy, vitamin B_12_ is supplied to the fetus by the mother through the placenta; the blood concentration of vitamin B_12_ is approximately twofold greater in the fetus than in the mother^27^. In addition, levels of vitamins B_6_, B_12_, and C in maternal blood have been reported to decrease during pregnancy^27^, which is consistent with our results. Because vitamins B_6_ and B_12_ are involved in homocysteine metabolism, the results of enrichment analysis for cluster 1 may reflect both maternal and fetal metabolism.

Using urinary metabolites, we successfully established a predictive model to estimate gestational age that exhibited performance equivalent to that of previously reported models involving plasma metabolites^11^. Therefore, gestational age could presumably be estimated from urinary metabolites. Because urine samples can be collected in a minimally invasive manner at each routine antenatal visit in most clinical settings, our results have potential clinical applications (e.g., in telemedicine using a medical examination kit).

Our predictive model mainly uses metabolites that demonstrate linear changes in levels during pregnancy. However, some metabolites demonstrate non-linear changes in levels (see Supplementary Fig. 1 online). For example, fatty acids play important roles in normal pregnancy; fatty acids reportedly accumulate in the mother during the first and second trimesters, then are released in the third trimester^28^. In the present study, many fatty acids were classified in cluster 2, such that they did not extensively contribute to the predictive model. However, the incorporation of metabolites with complex behaviors into our predictive model might enable more accurate prediction of gestational age.

Clinically, diagnosis of gestational weeks is conducted by using information regarding the last menstrual periods or fetal ultrasound findings at the early stage of pregnancy. This clinical information is sometimes inaccurate or inaccessible even in developed countries^29–31^. Therefore, estimation of gestational weeks by minimally invasive procedures is needed for various clinical settings in obstetrics, including determination of the due date, preterm birth and fetal growth. Taken together, our prediction model constructed by urinary metabolomics may provide useful clinical information for estimating gestational weeks in a variety of clinical settings.

The predicted gestational age was significantly older in cases who subsequently developed HDP. This result might be due to the presence of circulating metabolites caused by premature placental aging^32^. Premature placental aging associated with elevated oxidative stress and mitochondrial damages is known to cause placental insufficiency leading to HDP, including preeclampsia^32–34^. Urinary metabolite profiles also reportedly change with cellular senescence^35^. Therefore, the older predicted gestational age in the HDP cases, which implies aberrant acceleration of pregnancy, might be caused by changes in the urinary metabolite profile due to placental aging.

Importantly, we identified metabolites that were associated with the onset of HDP. For example, the increased level of 3-hydroxy-3-methylglutaric acid was significantly associated with the onset of HDP. In a rat model, 3-hydroxy-3-methylglutaric acid was able to induce physiological oxidative stress^36^. In addition, Nemeth et al. reported that the onset of HDP, including gestational hypertension, is related to increased maternal oxidative stress^37^. They indicated that insufficient capacity for glutathione recycling in patients with HDP might lead to reduced protection against oxidative stress. Furthermore, elevated oxidative stress is regarded as a manifestation of preeclampsia; for example, in the placentas of patients with preeclampsia, increased lipid peroxidation and decreased activities of some antioxidant enzymes have been identified^38^. Our results are suggestive of such physiological aberrations.

The increased levels of isocitric and urocanic acids also showed significant associations with HDP onset. Although isocitric acid is a component of the tricarboxylic acid cycle, the level of succinic acid (located downstream of isocitric acid) did not significantly differ between the healthy and HDP groups. This suggests reduced conversion of isocitric acid to oxalosuccinic acid, which requires nicotinamide adenine dinucleotide (NAD^+^). Therefore, the cellular NAD^+^ level may be reduced in cases who develop HDP^39^. Because NAD^+^ is required for DNA repair, DNA damage in the placenta caused by elevated oxidative stress associated with HDP might lead to a reduction of the NAD^+^/NADH ratio^40^. Urocanic acid is a histidine metabolite and a major component of ultraviolet light absorption in the skin. Its concentration in the plasma is reportedly elevated in cases with preeclampsia^41^, in agreement with our results.

In this study, we demonstrated that the predicted gestational age calculated by combining multiple metabolite levels could more accurately predict the onset of HDP, compared with each metabolite level alone (Fig. 5). This finding suggests that a combination of metabolites—each with weak explanatory power—could more accurately predict the risk, compared with individual metabolites; this was similar to the previously described polygenic risk score, which comprised the weighted sum of alleles associated with some traits^42^.

There were no significant differences in predicted gestational age or the levels of metabolites between the healthy and SPTB groups. Previous studies reported an association between urinary phthalate metabolites and SPTB^43,44^. Unfortunately, the metabolites could not be detected by gas chromatography-tandem mass spectrometry (GC–MS/MS) in our study. Although associations between maternal urinary or blood metabolites and SPTB development have rarely been described, various metabolites in cervicovaginal fluid, amniotic fluid, and neonatal urine are reportedly associated with SPTB^45–47^. SPTB often involves inflammatory changes caused by local bacterial infection in organs or tissues such as the vagina, decidua, placenta, and amniotic cavity^48^. Therefore, manifestations of SPTB may not be detected with urinary metabolites.

The present study had several limitations. First, all study participants were recruited at a single facility in Japan; therefore, our predictive model must be validated in independent facilities and in cohorts of patients with different ethnicities^49^. Second, we studied physiological changes in pregnant women solely on the basis of urinary metabolites; thus, comprehensive analyses that consider other omics data are warranted. In a previous prospective cohort study (i.e., the Maternity Log [MLOG] study), we obtained multi-omics information including the plasma metabolome, blood transcriptome, and urinary metabolome^49^. We hope that the validity of our predictive model will be verified by the analysis of relationships among urinary metabolites and other multi-omics data in the future. Third, the number of healthy pregnant women available for construction of the predictive model in this study was insufficient. In the TMM BirThree Cohort Study, which is a parent cohort of the MLOG study, 23,406 pregnant women were recruited^50,51^; moreover, urine samples were collected twice during pregnancy from each participant for use in the quantification of urinary metabolites. Urinary metabolite information from a very large number of pregnant women could further elucidate metabolomic changes during pregnancy; this might improve the predictive performance of our model. Finally, only relative quantification of metabolites was performed in this study. For metabolites that were found to have a significant association with the onset of HDP, we plan to increase the reliability of the evidence by absolute quantification in the near future.

In conclusion, we demonstrated dynamic changes in urinary metabolomic profiles of 184 metabolites during pregnancy, a predictive model was constructed by using urinary metabolite information to estimate gestational age at the time of urine specimen collection. The results suggested that urinary metabolite information is useful for understanding the normal progression of pregnancy, as well as for predicting the development of pregnancy complications. Minimally invasive urinary metabolomics might lead to breakthroughs in the analysis and management of healthy and complicated pregnancies in various clinical settings in the future.

## Methods

### Study setting

In total, 302 pregnant women were recruited at Tohoku University Hospital, Sendai, Japan, between September 2015 and November 2016; these women were previously included in the MLOG study^49^, which is an add-on study to the TMM BirThree Cohort Study^50,51^. Written informed consent was obtained from all participants by the genome medical research coordinators. The MLOG study was conducted under a collaborative research agreement among Tohoku Medical Megabank Organization, Tohoku University, and NTT DOCOMO, Inc. (Tokyo, Japan) and all procedures were in accordance with the Declaration of Helsinki. The study protocol was approved by the ethics committees of the Graduate School of Medicine (2014-1-704) and Tohoku Medical Megabank Organization (22017-1-085) at Tohoku University.

As shown in Supplementary Fig. 3 online, 187 healthy pregnant women were selected from the 302 recruited subjects; the data from these 187 women were used for the construction of a model to predict gestational age. Healthy pregnant women were defined as subjects who had a singleton pregnancy, gave birth at term, and did not develop any pregnancy complications. With respect to pregnancy complications observed in this study, 23 subjects developed HDP (excluding those with chronic hypertension) and 14 subjects gave SPTB. HDP was defined as gestational hypertension, preeclampsia, or superimposed preeclampsia, excluding chronic hypertension^52,53^. SPTB was defined as spontaneous labor at fewer than 37 weeks of gestation, excluding delivery by cesarean section.

### Urine collection

Either early morning first urine or spot urine specimen (10 mL) was collected at each antenatal visit; 12 samples were obtained from each subject. In total, 2741 urine samples were collected from healthy, HDP, or SPTB cases (2140 early morning first urine and 601 spot urine samples). All samples were stored at − 80 °C immediately after collection, then subjected to GC–MS/MS analysis.

### Chemical reagents

The following chemical reagents were used in this study: pyridine (Cat. No. Q003, Tokyo Chemical Industry Co. Ltd., Tokyo, Japan), methanol (Cat. No. 25185-76, Kanto Kagaku, Tokyo, Japan), chloroform (Cat. No. 07278-79, Kanto Kagaku), water (MilliQ, Millipore, Burlington, MA, USA), 2-isopropyl malic acid (Cat. No. 333115-100MG, Sigma-Aldrich, St. Louis, MO, USA), methoxyamine hydrochloride (Cat. No. 226904-25G, Sigma-Aldrich), and N-methyl-N-(trimethylsilyl) trifluoroacetamide (Cat. No. 1022-11061, GL Science, Saitama, Japan).

### GC–MS/MS analysis

To determine metabolite levels in maternal urine samples, GC–MS/MS analysis was performed using a previously described method^49,54^. The urine metabolites were extracted by a robotic system (Microlab STARlet Robot System, Hamilton, Reno, NV, USA). Fifty microliter of each urine sample was added to 260 μL of extraction solvent, consisting of chloroform/methanol/water/internal standard solution at a ratio of 1/2.5/1/0.18 (vol/vol/vol/vol); the internal standard solution contained 0.5 mg/mL 2-isopropylmalic acid. Each sample was shaken at 37 °C for 30 min, then centrifuged at 6231×g for 5 min at 4 °C. The supernatant was transferred to a 1.5-mL tube and lyophilized with a freeze-drying system (FDS-1000, EYELA, Tokyo, Japan). For the first derivatization, the residue was resuspended using 20 mg/mL methoxyamine hydrochloride in pyridine (80 μL) at 37 Hz for 20 min in an ultrasonic bath (UT-10, Sharp, Osaka, Japan); it was incubated at 1200 rpm for 90 min at 30 °C. After centrifugation at 16,000×g for 3 min at 4 °C, the supernatant (40 μL) was transferred to a glass vial and placed on the multifunctional autosampler system (AOC6000, Shimadzu, Kyoto, Japan). The metabolites in the sample were automatically derivatized with 20 μL of N-methyl-N-(trimethylsilyl)trifluoroacetamide using the multifunctional autosampler. The sample was then analyzed with gas chromatography triple quadrupole mass spectrometry (TQ-8040, Shimadzu). The sample (1 μL) was exposed to an injector (split ratio 30:1); the targeted analytes, including 2-isopropylmalic acid (internal standard), were separated using a capillary column (BPX5: 30-m length, 0.25-μm film thickness, 0.25-mm inner diameter; Cat. No. SGE054101, Shimadzu GLC Ltd., Tokyo, Japan) under helium gas with constant linear velocity of 39.0 cm/sec. The inlet temperature was set to 250 °C; the oven temperature was initially set to 60 °C for 2 min, then changed to 330 °C at a rate of 15 °C/min, and finally maintained at this temperature for 3 min. The electron ionization voltage, ion source temperature, and MS interface temperature were 70 eV, 200 °C, and 280 °C, respectively. The metabolites were identified with the Smart Metabolites Database (Shimadzu) and the peak area was calculated by Traverse MS (Reifycs, Tokyo, Japan). To adjust for measurement errors during each GC–MS/MS analysis, normalization was first conducted using 2-isopropylmalic acid as an internal standard added to each sample (see Supplementary Fig. 4 online). Normalization was repeated using quality control samples (see Supplementary Fig. 4 online) mixed second-trimester human urine (IR100040-Donor-HG, Innovative Research, Inc., Novi, MI, USA), third-trimester human urine (IR100041-DONOR-JT, Innovative Research, Inc.), and our study urine samples, which were injected at intervals of 12 study samples, in accordance with the reference quality control method^55^. Normalized levels of metabolites in the quality control samples were assessed by calculating coefficients of variation; metabolites with coefficients of variation > 20% were eliminated. Finally, 184 metabolites fulfilled the criteria of the second normalization. Thereafter, to avoid the dilution effect of urine, the levels of those metabolites were compensated by creatinine concentration in the samples measured by a biochemical test (Detaminer-L CRE, Hitachi Chemical Diagnostics Systems Co., Ltd., Tokyo, Japan).

### Data analysis

To investigate the profiles of urinary metabolites during normal pregnancy, we determined the average level of each metabolite according to gestational age in healthy pregnant women. The average levels were standardized for each metabolite by the z-score method, then subjected to hierarchical clustering analysis and trend analysis. The Ward variance minimization algorithm was selected for the clustering analysis^56^.

Subsequently, using the levels of urinary metabolites in healthy pregnant women, we constructed a predictive model to estimate gestational age at the time of urine collection. Elastic net regression was used for model construction, with the objective variable as the gestational age (days) and the explanatory variable as the logarithmic level of each of 184 metabolites. Elastic net regression has a substantial learning ability for features with multicollinearity, such as omics data^10^. The logarithms of metabolite levels were used to reduce the effects of outliers. Leave-one-out cross validation was performed; the data of one healthy pregnant woman were used as the test data, and after learning with the data of others, prediction was performed for the test data. This cycle was repeated according to the number of healthy pregnant women (n = 187). Then, the predictive performance of all combined test data was evaluated by the root mean squared error and Pearson correlation coefficient between actual and predicted gestational age.

Using the MBROLE 2.0 web-based analysis tool^57^, we performed enrichment analysis for 68 metabolites that consistently contributed to the prediction; no metabolite coefficient in the model reached zero during 187 cycles in the leave-one-out cross validation. Enrichment analysis was performed for each metabolite cluster, targeting the biofunctions defined by Human Metabolome Database^58^.

Finally, by entering urinary metabolite data from HDP or SPTB cases into the model constructed by data from all healthy pregnant women in this study, we examined whether outputs significantly differed between healthy and complicated pregnancies.

For 68 metabolites, we also identified those that were associated with the onset of HDP. The association between the onset and each metabolite according to gestational age was confirmed by using the Wald test for logistic regression, with the onset of HDP as the objective variable (i.e., healthy = 0 and HDP = 1) and the level of each metabolite as the explanatory variable.

Principal component analysis (PCA) of 2741 urine samples revealed significant differences between early morning first urine and spot urine samples with respect to both first and second principal components (see Supplementary Figs. 5 and 6 online). Therefore, when constructing our predictive model for gestational age, we used the levels of urinary metabolites in healthy pregnant women, as well as their urine sample types (i.e., early morning urine = 0 and spot urine = 1). In hierarchical clustering analysis, we used only early morning urine samples; these are less likely to be affected by each subject’s lifestyle or behavior. Furthermore, we found a significant difference in maternal age between healthy and HDP cases (Table 1). Therefore, to exclude the effects of maternal age and urine type, we evaluated the association between each metabolite and the onset of HDP by using the Wald test for logistic regression, with maternal age and urine type as covariances. Considering the number of metabolites (n = 68), we used the Benjamini–Hochberg method to compensate for multiple Wald tests. Metabolites with a false discovery rate of less than 0.05 at any one gestational week were defined as those significantly associated with HDP.

Python libraries as follows; (1) Seaborn^59^, (2) Scikit-learn^60^, and (3) Statsmodels^61^, were used for (1) the hierarchical clustering analysis and each figure visualization, (2) the trend analysis, predictive model construction and PCA, and (3) the Wald test for logistic regression, respectively.

Data are presented as the mean ± S.D. (standard deviation) unless otherwise indicated. Computational resources were provided by the Tohoku Medical Megabank Organization supercomputer system.

## Supplementary Information

Supplementary Information.
