# Supplementary material for: Machine learning approaches to predict gestational age in normal and complicated pregnancies via urinary metabolomics analysis
Source: Sci Rep. 2021 Sep 7;11:17777. doi: 10.1038/s41598-021-97342-z (PMC8423760; doi:10.1038/s41598-021-97342-z)
Supplement: Supplementary file 1 — Supplementary Information. [file 41598_2021_97342_MOESM1_ESM.pdf]

Supplementary Information

Machine learning approaches to predict gestational age in normal and complicated pregnancies via urinary metabolomics analysis

Takafumi Yamauchi, Daisuke Ochi, Naomi Matsukawa, Daisuke Saigusa, Mami Ishikuro, Taku Obara, Yoshiki Tsunemoto, Satsuki Kumatani, Riu Yamashita, Osamu Tanabe, Naoko Minegishi, Seizo Koshiba, Hirohito Metoki, Shinichi Kuriyama, Nobuo Yaegashi, Masayuki Yamamoto, Masao Nagasaki, Satoshi Hiyama, and Junichi Sugawara*

*Corresponding author:

Junichi Sugawara, M.D., Ph.D.

Tohoku Medical Megabank Organization, Tohoku University, 2-1 Seiryo-machi, Aoba-ku, Sendai 980-8573, Japan

E-mail: jsugawara@med.tohoku.ac.jp

Tel: +81-22-273-6283

Fax: +81-22-273-6410

Supplementary Fig. 1: Trend analysis for 184 metabolites of healthy pregnant women. (A), (B) and (C) indicate the trends of metabolites in clusters 1 (n = 93), 2 (n = 49), and 3 (42), respectively. The red lines show levels of metabolites in urine samples from 187 healthy pregnant women were averaged for each gestational week, standardized by the z-score method. The blues are the fitting curves by the quadratic function for each metabolite.

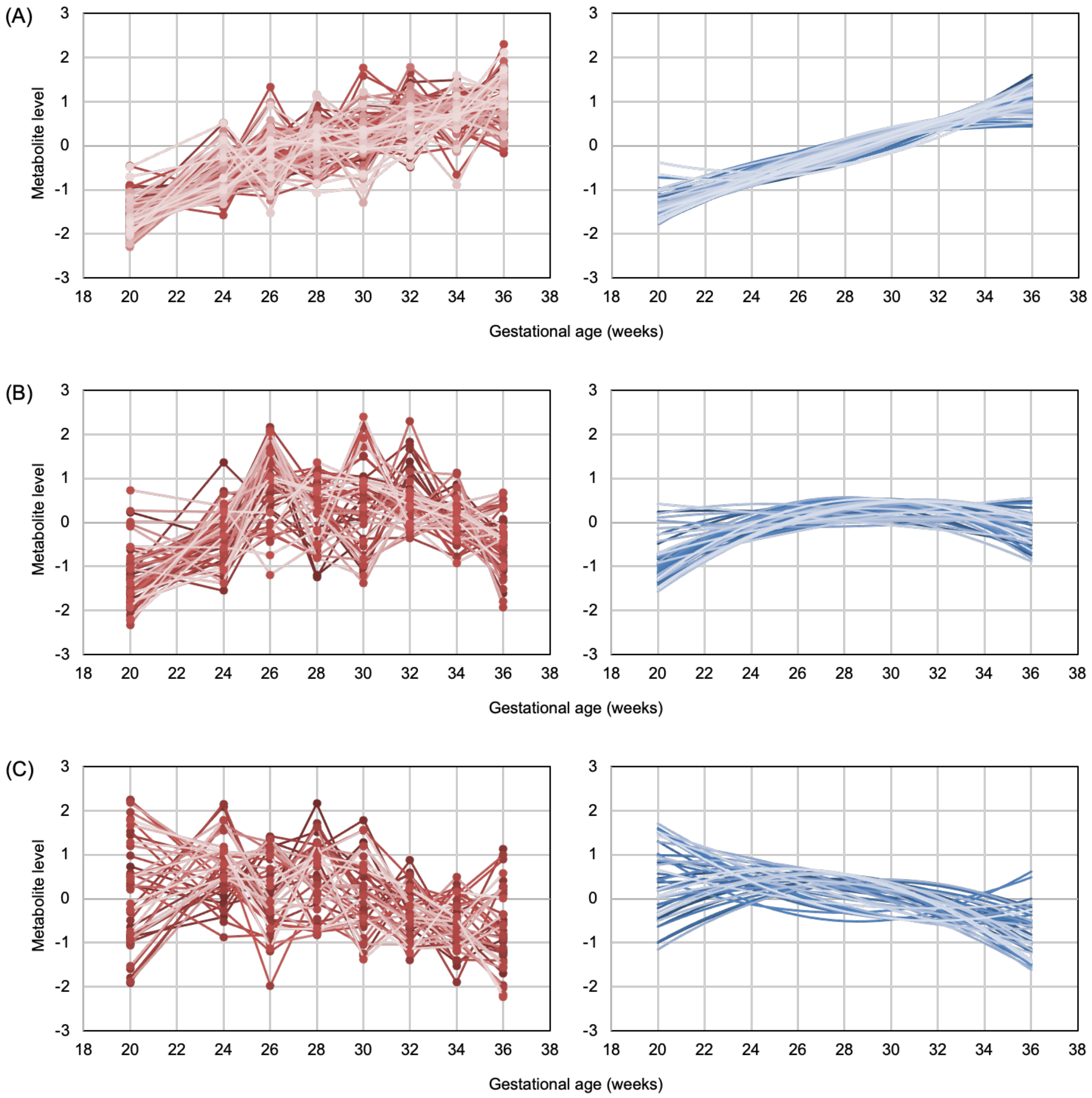

Supplementary Fig. 2: Comparison of predicted gestational ages between two age groups of healthy pregnant women. Blue and orange boxes indicate younger (< 35 years) and older (≥ 35 years) age groups, respectively. There was no statistically significant difference in predicted gestational age between the two groups, according to analysis with Welch's t-test ($p < 0.05$).

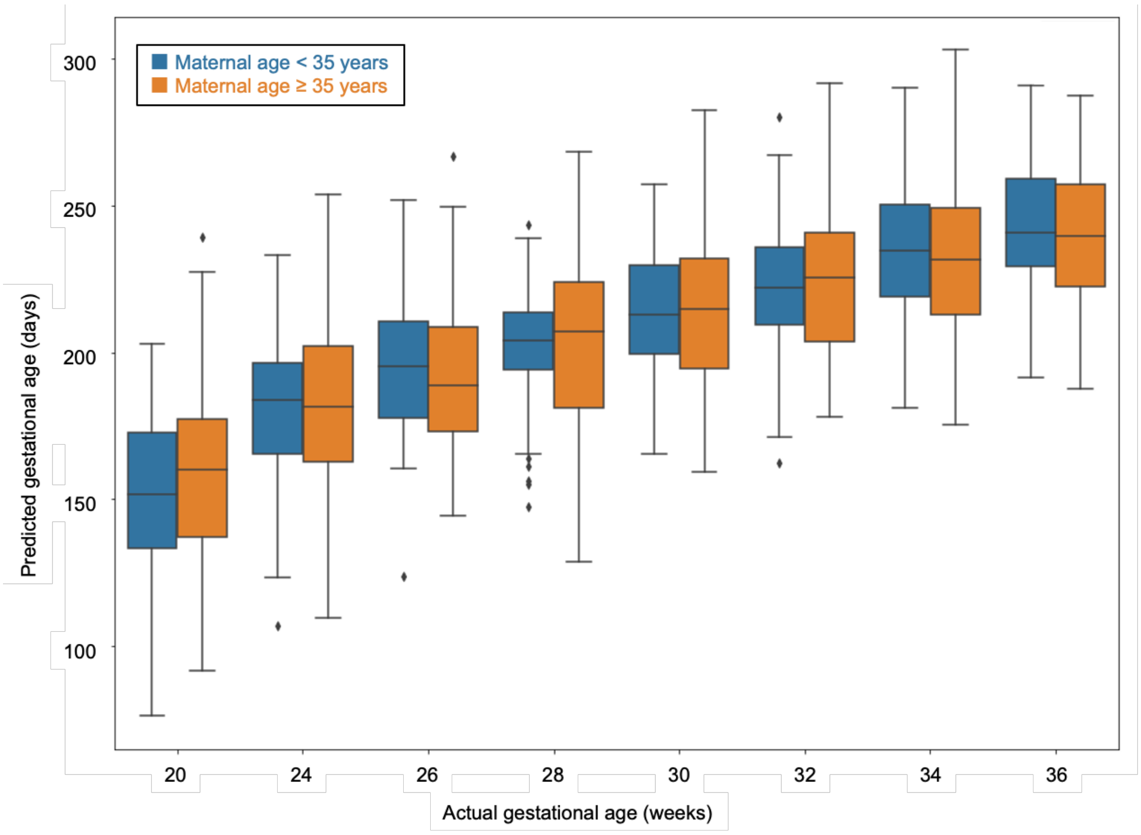

Supplementary Fig. 3: Selection of study subjects. In total, 187 healthy pregnant women were selected from the 302 recruited subjects. Healthy pregnant women were defined as subjects who had a singleton pregnancy, gave birth at term, and did not develop any pregnancy complications. With respect to pregnancy complications observed in this study, 23 subjects developed HDP (excluding those with chronic hypertension) and 14 subjects gave SPTB.

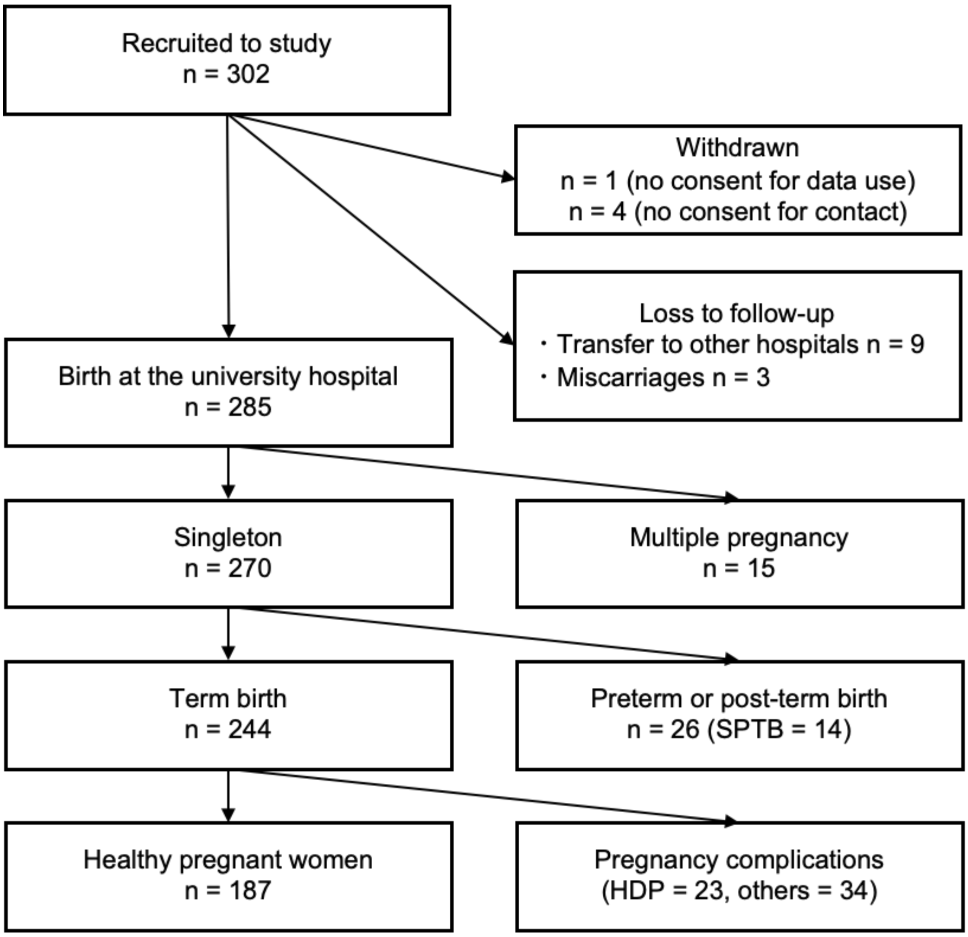

Supplementary Fig. 4: Normalization by internal standards and quality control samples for levels of creatinine. Dots and error bars indicate mean and standard deviation of creatinine peak area (PA) for each sample injection order, respectively, and blue for subject's samples, red for quality control (QC) samples. (A) PA before normalization, (B) PA after internal standard (IS) normalization (each PA / each IS × mean of all IS), and (C) PA after IS and QC sample normalization.

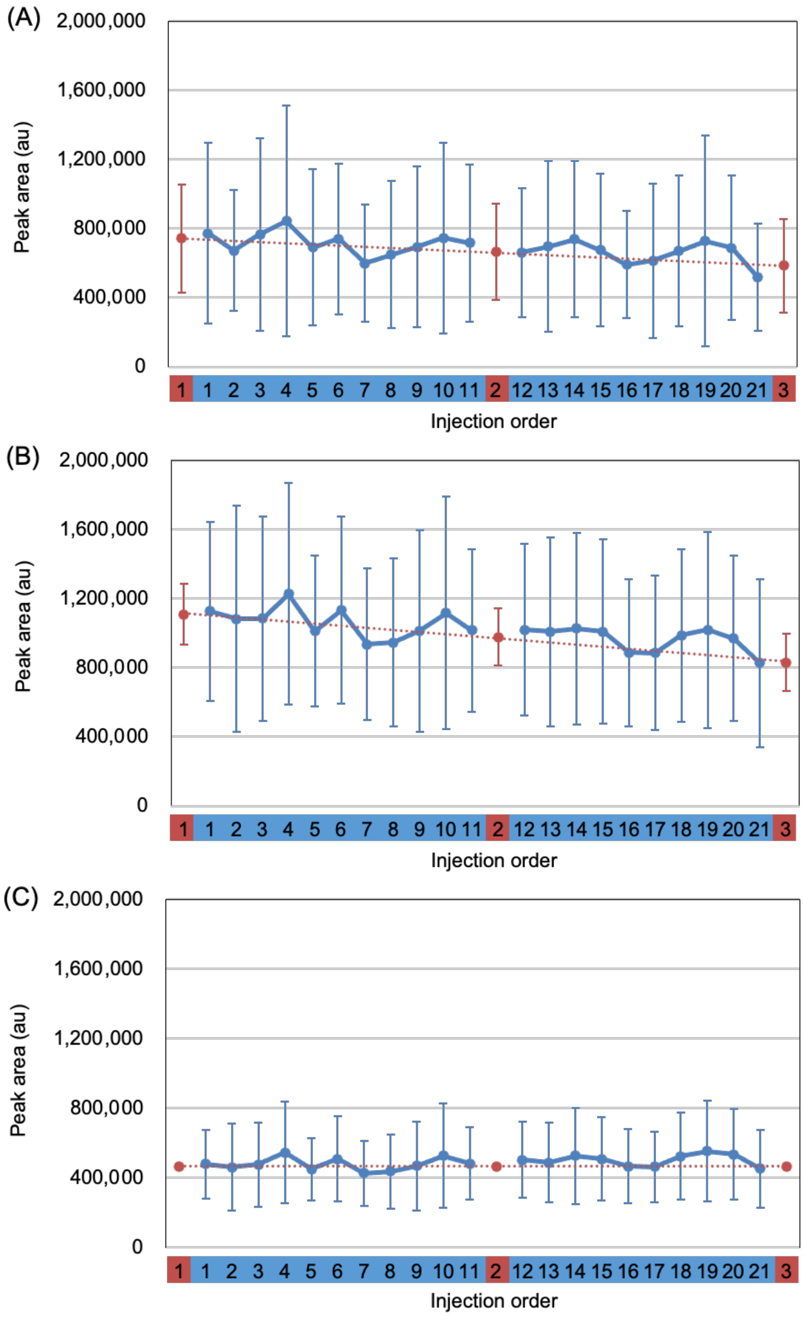

Supplementary Fig. 5: Principal components analysis plot of 2,741 urine samples.

Red and blue dots indicate early morning first urine (n = 2,140) and spot urine (n = 601) samples, respectively. PC1 and PC2 represent first and second principal components, respectively.

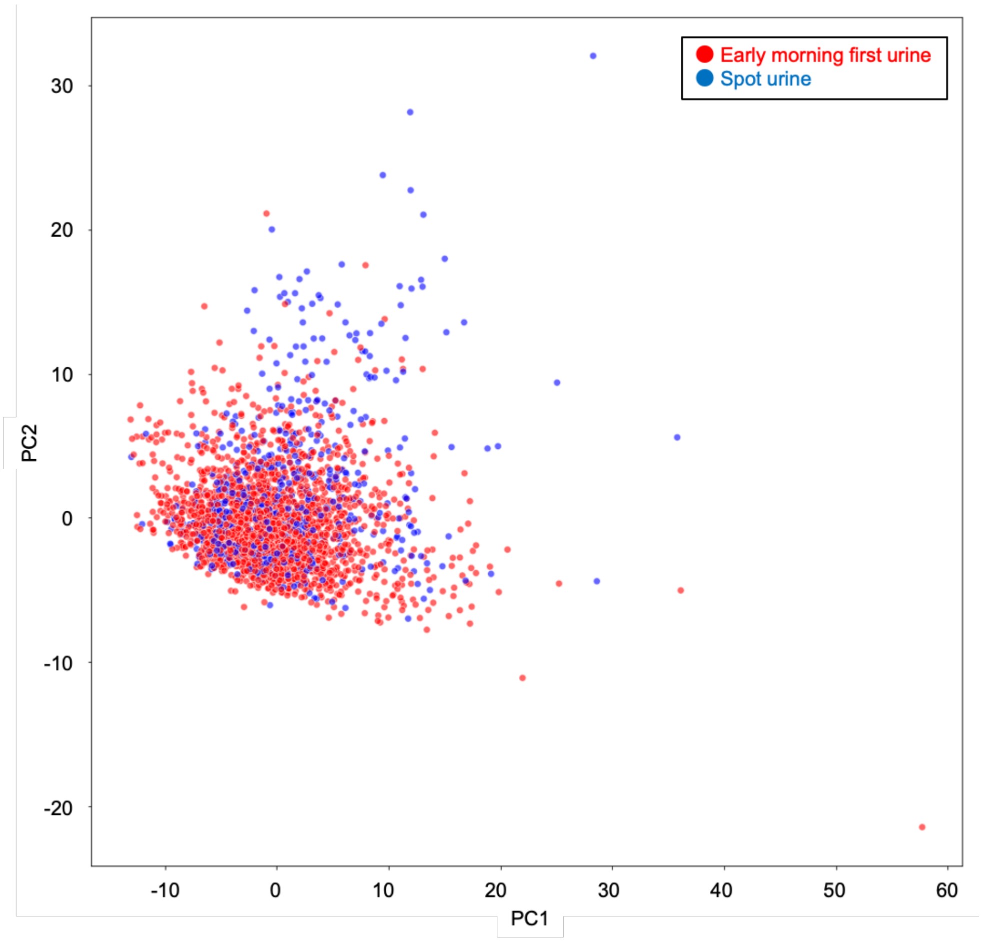

Supplementary Fig. 6: Comparison of principal components in principal components analysis between two types of urine samples. PC1 (A) and PC2 (B) of early morning first urine and spot urine samples are shown as boxplots. Significant differences in principal components were observed between the two types of urine samples by Welch's t-test ($p < 0.05$).

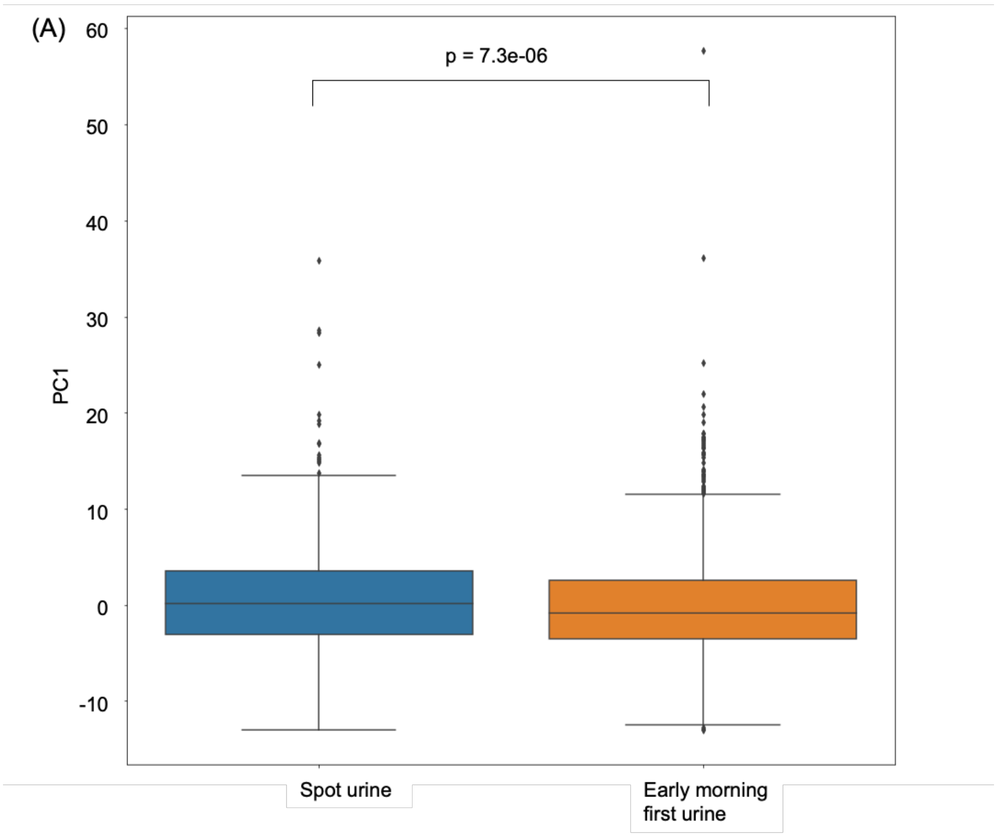

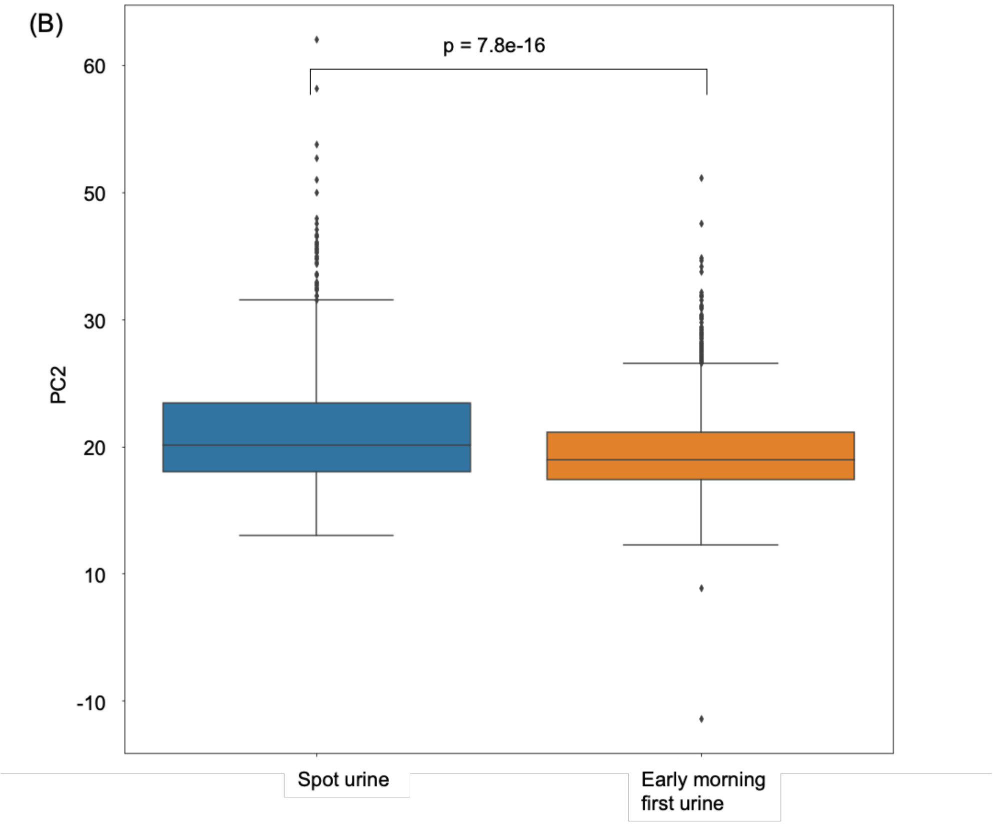
(B)
p = 7.8e-16
PC2
60
50
30
20
10
-10
Spot urine
Early morning
first urine

Supplementary Table 1: Information on clusters and chemical families of 184 compounds, and whether they correspond to the 68 metabolites that have contributed to the predictive model.

Metabolite	Cluster	Chemical family*	Plus / minus sign of coefficient
3-Hydroxyphenylacetic acid	1	1-hydroxy-4-unsubstituted benzenoids	- (not included in 68 metabolites)
Monostearin	1	1-monoacylglycerols	-
2-Aminoethanol	1	1,2-aminoalcohols	P (plus)
Adenine	1	6-aminopurines	-
N-Acetylmannosamine	1	Acylaminosugars	P
Alanine	1	Alanine and derivatives	-
Kynurenine	1	Alkyl-phenylketones	M (minus)
2-Aminopimelic acid	1	Alpha amino acids	M
2-Aminoisobutyric acid	1	Alpha amino acids	-
Dimethylglycine	1	Alpha amino acids	-
Sarcosine	1	Alpha amino acids	-
Lactic acid	1	Alpha hydroxy acids and derivatives	P
Glycolic acid	1	Alpha hydroxy acids and derivatives	-
Pyruvic acid	1	Alpha-keto acids and derivatives	-
Ureidosuccinic acid	1	Aspartic acid and derivatives	P
N-Acetylaspartic acid	1	Aspartic acid and derivatives	-
3-Hydroxyisobutyric acid	1	Beta hydroxy acids and derivatives	-
3-Hydroxypropionic acid	1	Beta hydroxy acids and derivatives	-
3-Hydroxybutyric acid	1	Beta hydroxy acids and derivatives	-
3-Hydroxyglutaric acid	1	Beta hydroxy acids and derivatives	-
Malic acid	1	Beta hydroxy acids and derivatives	-
Ethylmalonic acid	1	Branched fatty acids	P
Dopamine	1	Catecholamines and derivatives	-
Inositol	1	Cyclohexanols	P
Cysteine	1	Cysteine and derivatives	M
Spermine	1	Dialkylamines	P

Succinic acid	1	Dicarboxylic acids and derivatives	M
Maleic acid	1	Dicarboxylic acids and derivatives	M
Glutaric acid	1	Dicarboxylic acids and derivatives	M
Methylmalonic acid	1	Dicarboxylic acids and derivatives	–
Fumaric acid	1	Dicarboxylic acids and derivatives	–
Malonic acid	1	Dicarboxylic acids and derivatives	–
2-Ketoglutaric acid	1	Gamma-keto acids and derivatives	M
Glucaric acid	1	Glucuronic acid derivatives	P
Galacturonic acid	1	Glucuronic acid derivatives	–
Glucuronic acid	1	Glucuronic acid derivatives	–
Glutamic acid	1	Glutamic acid and derivatives	–
Glucose 6-phosphate	1	Hexose phosphates	–
Fucose	1	Hexoses	–
Glucose	1	Hexoses	–
Rhamnose	1	Hexoses	–
Hydroxylamine	1	Homogeneous other non-metal compounds	–
3-Hydroxy-3-methylglutaric acid	1	Hydroxy fatty acids	P
2-Hydroxyisocaproic acid	1	Hydroxy fatty acids	P
2-Hydroxyisovaleric acid	1	Hydroxy fatty acids	–
Citramalic acid	1	Hydroxy fatty acids	–
3-Hydroxyanthranilic acid	1	Hydroxybenzoic acid derivatives	–
7-Methylguanine	1	Hypoxanthines	P
Urocanic acid	1	Imidazolyl carboxylic acids and derivatives	P
Threonine	1	L-alpha-amino acids	P
Methionine sulfone	1	L-alpha-amino acids	P
Homoserine	1	L-alpha-amino acids	–
Adipic acid	1	Medium-chain fatty acids	M
Decanoic acid	1	Medium-chain fatty acids	–
Nonanoic acid	1	Medium-chain fatty acids	–
Octanoic acid	1	Medium-chain fatty acids	–
Methionine	1	Methionine and derivatives	–

Homovanillic acid	1	Methoxyphenols	P
Coniferyl alcohol	1	Methoxyphenols	–
3–Methylglutaric acid	1	Methyl–branched fatty acids	P
Batyl alcohol	1	Monoalkylglycerols	P
Xylulose	1	Monosaccharides	–
Sorbose	1	Monosaccharides	–
Tiglylglycine	1	N–acyl–alpha amino acids	–
Niacinamide	1	Nicotinamides	P
Lactose	1	O–glycosyl compounds	–
Ribonolactone	1	Pentoses	P
Ribose	1	Pentoses	M
Ribulose	1	Pentoses	–
4–Hydroxyphenyllactic acid	1	Phenylpropanoic acids	–
4–Hydroxyphenylpyruvic acid	1	Phenylpyruvic acid derivatives	–
Xanthosine	1	Purine nucleosides	M
Adenosine	1	Purine nucleosides	–
Quinolinic acid	1	Pyridinecarboxylic acids	P
Nicotinic acid	1	Pyridinecarboxylic acids	–
Dihydrouracil	1	Pyrimidones	P
Serine	1	Serine and derivatives	–
2–Hydroxyglutaric acid	1	Short–chain hydroxy acids and derivatives	P
Oxalacetic acid	1	Short–chain keto acids and derivatives	P
3–Methyl–2–oxovaleric acid	1	Short–chain keto acids and derivatives	M
2–Ketoisocaproic acid	1	Short–chain keto acids and derivatives	–
Glyceric acid	1	Sugar acids and derivatives	P
Ribonic acid	1	Sugar acids and derivatives	–
Threitol	1	Sugar alcohols	–
Xylitol	1	Sugar alcohols	–
Isocitric acid	1	Tricarboxylic acids and derivatives	P
Aconitic acid	1	Tricarboxylic acids and derivatives	–
Citric acid	1	Tricarboxylic acids and derivatives	–
Tryptamine	1	Tryptamines and derivatives	–
Ureidopropionic acid	1	Ureas	–

3-Aminoglutaric acid	1	- (not annotated in the Human Metabolome Database)	-
3-Dehydroshikimic acid	1	-	-
3-Methylcrotonoylglycine	1	-	-
Glycine	2	Alpha amino acids	M
2-Aminoadipic acid	2	Alpha amino acids	-
Creatinine	2	Alpha amino acids and derivatives	-
Asparagine	2	Asparagine and derivatives	-
3-Aminoisobutyric acid	2	Beta amino acids and derivatives	-
Glyoxylic acid	2	Carboxylic acids	-
5-Aminolevulinic acid	2	Delta amino acids and derivatives	M
5-Aminovaleric acid	2	Delta amino acids and derivatives	M
Glucono-1,5-lactone	2	Gluconolactones	-
Lyxose	2	Glucuronic acid derivatives	-
N-Acetylglutamine	2	Glutamine and derivatives	-
3-Hydroxyisovaleric acid	2	Hydroxy fatty acids	-
Tryptophan	2	Indolyl carboxylic acids and derivatives	-
Arginine	2	L-alpha-amino acids	M
3-Sulfinoalanine	2	L-alpha-amino acids	M
Glutamine	2	L-alpha-amino acids	-
Cystathionine	2	L-cysteine-S-conjugates	M
Myristic acid	2	Long-chain fatty acids	-
Palmitic acid	2	Long-chain fatty acids	-
Stearic acid	2	Long-chain fatty acids	-
Azelaic acid	2	Medium-chain fatty acids	-
Pimelic acid	2	Medium-chain fatty acids	-
Caproic acid	2	Medium-chain fatty acids	-
Suberic acid	2	Medium-chain fatty acids	-
Vanilmandelic acid	2	Methoxyphenols	M
Mesaconic acid	2	Methyl-branched fatty acids	M
Methylsuccinic acid	2	Methyl-branched fatty acids	-
Putrescine	2	Monoalkylamines	-
Ribulose 5-phosphate	2	Monosaccharide phosphates	-
Acetylglycine	2	N-acyl-alpha amino acids	M

Isovalerylglycine	2	N-acyl-alpha amino acids	-
Isobutyrylglycine	2	N-acyl-alpha amino acids	-
N-Acetylserine	2	N-acyl-L-alpha-amino acids	-
Arabinose	2	Pentoses	-
Tyramine	2	Phenethylamines	-
Phenylalanine	2	Phenylalanine and derivatives	M
Guanine	2	Purines and purine derivatives	P
Pyridoxamine	2	Pyridoxamine 5'-phosphates	-
Orotic acid	2	Pyrimidinecarboxylic acids	-
2-Ketobutyric acid	2	Short-chain keto acids and derivatives	-
Acetoacetic acid	2	Short-chain keto acids and derivatives	-
Gluconic acid	2	Sugar acids and derivatives	M
Tartaric acid	2	Sugar acids and derivatives	M
meso-Erythritol	2	Sugar alcohols	-
Tyrosine	2	Tyrosine and derivatives	-
Dopa	2	Tyrosine and derivatives	-
Metoprolol	2	Tyrosols and derivatives	-
Uric acid	2	Xanthines	-
Threo-b-hydroxyaspartic acid	2	-	-
4-Hydroxyphenylacetic acid	3	1-hydroxy-2-unsubstituted benzenoids	-
5-Oxoproline	3	Alpha amino acids and derivatives	M
2-Hydroxyisobutyric acid	3	Alpha hydroxy acids and derivatives	M
2-Hydroxybutyric acid	3	Alpha hydroxy acids and derivatives	-
Benzoic acid	3	Benzoic acids	-
Ascorbic acid	3	Butenolides	P
Catechol	3	Catechols	-
Oxalic acid	3	Dicarboxylic acids and derivatives	P
5-Hydroxymethyl-2-furoic acid	3	Furoic acids	-
Glycerol 3-phosphate	3	Glycerophosphates	-
Glucosamine	3	Hexoses	M
Psicose	3	Hexoses	M
Hippuric acid	3	Hippuric acids	-

2-Hydroxyhippuric acid	3	Hippuric acids	–
Histidine	3	Histidine and derivatives	–
Hydroquinone	3	Hydroquinones	–
2-Methyl-3-hydroxybutyric acid	3	Hydroxy fatty acids	M
4-Hydroxybenzoic acid	3	Hydroxybenzoic acid derivatives	–
Protocatechuic acid	3	Hydroxybenzoic acid derivatives	–
Thymine	3	Hydroxypyrimidines	M
Hypoxanthine	3	Hypoxanthines	–
Allantoin	3	Imidazoles	–
Indol-3-acetic acid	3	Indole-3-acetic acid derivatives	–
N6-Acetyllysine	3	L-alpha-amino acids	M
Lysine	3	L-alpha-amino acids	–
Cystine	3	L-cysteine-S-conjugates	M
3-Methoxy-4-hydroxybenzoic acid	3	M-methoxybenzoic acids and derivatives	M
Dihydroxyacetone phosphate	3	Monosaccharide phosphates	–
Phosphoric acid	3	Non-metal phosphates	–
3-Phenyllactic acid	3	Phenylpropanoic acids	M
O-Phosphoethanolamine	3	Phosphoethanolamines	M
Pantothenic acid	3	Polyols	M
Cytosine	3	Pyrimidones	–
Uracil	3	Pyrimidones	–
Kynurenic acid	3	Quinoline carboxylic acids	–
Threonic acid	3	Sugar acids and derivatives	M
Arabitol	3	Sugar alcohols	P
Glycerol	3	Sugar alcohols	–
N-Acetyltyrosine	3	Tyrosine and derivatives	–
Xanthine	3	Xanthines	M
ParaXanthine	3	Xanthines	M
Pyrogallol	3	–	M

* The chemical family indicates the "Direct parent" defined by the Human Metabolome Database.

Supplementary Table 2: Urinary metabolites associated with HDP onset.

Metabolite	Gestational weeks	Coefficient of logistic regression	*p*-value	False discovery rate
Isocitric acid	28	0.828	0.0013	0.044
Urocanic acid	28	13.8	0.00087	0.044
3-Hydroxy-3-methylglutaric acid	34	0.849	0.00062	0.021
Isocitric acid	34	1.12	0.000037	0.0025
